# ‘We need someone to deliver our voices’: reflections from conducting remote qualitative research in Syria

**DOI:** 10.1186/s13031-021-00361-w

**Published:** 2021-04-17

**Authors:** Yazan Douedari, Mervat Alhaffar, Diane Duclos, Mohamed Al-Twaish, Samer Jabbour, Natasha Howard

**Affiliations:** 1 Syria Research Group (SyRG), co-hosted by the London School of Hygiene and Tropical Medicine London, UK; and Saw Swee Hock School of Public Health, Singapore, Singapore; 2 London School of Hygiene and Tropical Medicine, 15-17 Tavistock Place, London, WC1H 9SH UK; 3 Hand in Hand for Aid and Development / İyilik için el ele derneği, Idlib, Syria; 4 American University of Beirut, Faculty of Health Sciences, Riad El Solh, Beirut, 1107 2020 Lebanon; 5 Saw Swee Hock School of Public Health, National University of Singapore, 12 Science Drive 2, Singapore, 117549 Singapore

**Keywords:** Remote research, Participatory methods, Online interviews, Research decolonisation, Syria

## Abstract

The need to generate evidence in spaces considered insecure and inhabited by potentially extremely vulnerable individuals (e.g. conflict-affected people who may not have means to move) has led researchers to study conflict-affected settings remotely. Increased attention to remote research approaches from social scientists, due to COVID-19-related travel restrictions, is sparking interest on appropriate methods and tools. Drawing on several years’ experience of remotely conducting qualitative research in Syria, we discuss challenges and approaches to conducting more inclusive, participatory, and meaningful research from a distance. The logistics, ethics, and politics of conducting research remotely are symptomatic of broader challenges in relation to the decolonisation of global and humanitarian health research. Key to the success of remote approaches is the quality of the relationships researchers need to be able to develop with study participants without face-to-face interactions and with limited engagement ‘in the field’. Particularly given overdue efforts to decolonise research institutions and methods, lead researchers should have a meaningful connection with the area in which they are conducting research. This is critical both to reduce chances that it will be extractive and exploitative and additionally for the quality of interpretation.

## Background

Conducting rigorous qualitative research during conflict is necessary for generating greater contextual knowledge of humanitarian crises, including the health consequences of violence and how conflict-affected communities survive political, social, and economic destruction. However, the practical and ethical challenges involved in implementing research in these settings – constrained access to populations of concern, building trust, risks for researcher and participant integrity, problematisation of ‘local ethics approvals’ when governments are targeting civilians – can discourage robust efforts. Therefore, humanitarian and conflict studies fields tend to ‘over-research’ groups such as refugees in what are considered ‘easier to reach’ settings [1], while overlooking those considered ‘harder to reach’, whose specific vulnerabilities may have prevented their relocation. The very categories of ‘lack of access’ and ‘hard-to-reach’ have arisen from the domination of the ‘foreign gaze’ in humanitarian studies [2], and must be used cautiously. We therefore need to critique and adapt our research methods and constantly examine researcher privilege, including how global and ‘local’ perspectives shape empirical and theoretical endeavours. Reflecting on the use of qualitative remote research in conflict-affected settings offers opportunities to address these issues.

Across the diverse studies our research team has implemented remotely in Syria, participants repeatedly highlighted the imperative to ‘*paint the full picture*’ and ‘*deliver our voices*’, forcefully arguing for untold stories to travel and be heard and radical optimism that positive change is possible. How we attempt to assemble such ‘painting’ and amplification from a distance is a complex and discursive process that requires unpacking. Whose voices are being amplified, how, by whom and for whom? Accounting for the multiple perspectives of people living in uncertain humanitarian environments cannot be achieved without in-depth engagement and continued examination not only of the rapidly-changing dynamics of conflicts, but also of the more mundane power dynamics structuring the societies studied, including intersectional (e.g. gendered, socioeconomic, racialized) inscriptions of power [3]. For example, our research in Syria requires knowledge of its fractured areas of control and actors, health systems governance approaches, and humanitarian system responses, so as to assess the potential and limitations of the amplification of personal narratives to trigger change. Remote research therefore compels us to think about engagements with ‘the field’ across and beyond the ‘being there’ stance of qualitative research. In other words, conversations on the remoteness of methods need to be embedded in broader conversations on the lenses that we use to frame and investigate issues as well as reflections on researchers’ positionalities [4, 5].

Syria has experienced ten years of conflict triggered by violent government responses to peaceful protests in 2011, and is divided into several areas of military control with fragmented governance and health systems struggling to respond to the impact of conflict and now the COVID-19 pandemic [6, 7]. The need to generate information in spaces that are difficult to access and inhabited by potentially very vulnerable people (e.g. those without means to move) pushed researchers to study public health in Syria from outside the country [8]. COVID-19 travel restrictions have sparked increased interest from social scientists in appropriate methods for remote research [9, 10]. This provides an opportunity to discuss more inclusive and participatory ways of conducting meaningful research from a distance. More importantly, the logistics, politics, and ethics of conducting research remotely are symptomatic of broader needs to decolonise global health and humanitarian studies. We offer to this debate lessons and reflections from five years of remote qualitative research in Syria. Completed and ongoing studies we draw from include research on health systems governance across Syria, exploration of women’s lived experiences of health services, discrimination, and gender-based violence during the Syrian conflict, COVID-19 response governance across Syria, and examination of responses to COVID-19 among displaced communities in opposition-controlled northwest Syria [11, 12].

### Why conduct qualitative research remotely in conflict settings?

In their book on research methods in conflict-affected settings, Mazurana and colleagues identify three groups that conflict-affected populations must accommodate: (i) armed combatants, (ii) professional humanitarian industry staff, and (iii) observers or witnesses, including researchers, who can contribute to ‘enabling or obstructing peace’ [13]. There is a strong ethical justification to conduct research in conflict-affected settings [14], including to document the direct and indirect consequences of armed conflicts on population health and to inform culturally-appropriate and conflict-sensitive health interventions aligned with the priorities of affected communities. Operational research between academic institutions and humanitarian organisations is also needed to provide evidence-based interventions to meet the needs of conflict affected populations and humanitarian responders – including health providers – in crises [15]. Qualitative research is particularly well placed to account for lived experiences of war and aid delivery, and to counter hegemonic narratives of crises [16–19]. Researchers working in conflict-affected settings have been grappling with the possibilities and impossibilities of conducting ‘field research’ for decades [20]. The opportunities and challenges stemming from innovative approaches developed over time by local, indigenous, diaspora, and international researchers can inform the broader research community, currently facing unprecedented challenges from the COVID-19 pandemic in conducting face-to-face research [21, 22]. Amongst these innovations, remote data collection methods – whether to complement ongoing studies or explore new research domains – are gaining academic attention.

Remote research is defined as any research in which participants and researchers do not interact in-person [23]. It is typically conducted via phone or online communications software (e.g. WhatsApp), but can include written, audio, or visual material generated by participants, such as photovoice or platforms allowing voices from conflict to be heard by global audiences (https://www.healthworkersatthefrontline.org/). Remote strategies are not suited for all research. Similar to other approaches relying on digital technologies to access and interview participants, remote research can potentially further exclude certain categories of individuals – commonly children and the elderly have been more difficult to represent [24]. Furthermore, digital research encounters affect the rapport built between researchers and study participants [25]. Remote data collection can reify conflict dynamics, especially when co-opted by elite actors [26]. However, it can also facilitate inclusion of often marginalised voices (e.g. homebound people – whether due to disability or caring responsibilities, those in remote or insecure settings). Using remote approaches in conflict-affected settings can enable wider and sometimes competing perspectives to converse, particularly when researchers harness local networks and forms of engagement. For example, we conducted remote interviews in three different military-controlled areas in Syria, without travelling from one area to another and talking to participants in places and times suitable for them, reducing potential security risks for all. Two major benefits of this were reducing risk and representing diversity. First, current literature often treats Syria as a homogeneous whole, without accounting for pre-conflict provincial diversities or regional differences imposed by conflict [27–30]. Second, it is not feasible for the same researcher to conduct in-person interviews in more than one area-of-control, as travelling between areas is neither tolerated nor safe.

### Five reflective criteria for assessing whether remote methods may be appropriate

Five essential criteria are emerging from our work, which can be used to reflect on the appropriateness and feasibility of conducting remote research: (i) research question and objectives; (ii) target participants; (iii) data collection budget and timeframe; (iv) research ethics and (v) community engagement.

i. *Appropriateness of remote methods to answer your research questions.* While the feasibility of research may guide, to some extent, the methodological approach that will be implemented in a conflict-affected setting, considering whether the methods align with research question and objectives are critical. For example, if a research question is best answered by ethnographic methods, remote interviews, similarly to face-to-face key informant interviews, may be useful initially but are unlikely to suffice for the whole study [31].
ii. *Appropriateness of remote methods to reach your target research participants*. It is important to consider first if target participants live in areas with good internet connexion, have access to necessary communication technologies, and are comfortable using them. For example, remote methods will likely work for young professionals or students, as they tend to be more comfortable with conference calls, online meetings, and screen sharing. However, participants who are older or with poorer internet access and skills could find remote approaches challenging (e.g. particularly more complex activities such as ‘unmute’ or ‘raise hand’). In such cases, if ethics approvals allow, it can work for the participant to get help from a younger relative or friend who is comfortable with the technology. Second, consider the possibility of developing relationships with research participants remotely, in a meaningful and ethical way. For example, drawing on pre-existing professional and personal networks can help foster new relationships remotely. Following-up with research participants who were involved in past data collection (e.g. through longitudinal research designs, transcript and findings review, and dissemination engagement activities) can increase comfort and trust in interactions. Third, assess how your remote approach may exclude certain participants and perpetuate inequalities and whether there are ways to mitigate this [32].
iii. *Potential of remote methods to help you overcome financial constraints.* While this should never be the primary consideration, it can be challenging to raise funds for research in insecure conflict-affected places. Funders can be reluctant to support research in settings where research designs that are considered gold-standard cannot be implemented, or where high levels of uncertainty require flexible approaches to data collection and incremental or less visible outcomes. Remote research is typically more cost-effective, trading travel costs for software costs. This can be helpful in many situations, such as conducting interviews in multiple rounds over time, in regions controlled by different groups, or in multiple countries.
iv. *Compliance with research standards and ethical requirements and institutional review norms.* Critical engagement with ethics review norms is crucial for remote research in conflict-affected settings. However, particularly in conflict-affected settings, formal engagements with review boards controlled by ‘illegitimate’ or contested authorities can potentially put research participants at risk. It is therefore important to consider alternative ways of engaging research participants if necessary in the design of your research approach and tools.
v. *Compatibility of remote methods with community engagement within your research design.* Understanding how community engagement speaks to local realities requires nuanced understanding of contexts and cannot be approached as a ‘box ticking’ exercise. Researchers need to assess the implications of remote engagement with individuals and communities, to ensure they are not putting anyone at additional risk or reinforcing unbalanced power structures (e.g. by reproducing judgemental or exclusionary dynamics). Having informal conversations with a broad range of potential participants and stakeholders before designing and implementing research or publishing findings can help both engage and protect study participants. Closely and regularly monitoring societal debates on social media can prove useful, especially in settings where access to public media is hindered by repression and violence.

### Methodological considerations for each remote research stage

Despite its benefits, remote qualitative research is not the norm and many social scientists still have limited experience with its logistical and ethical considerations. We thus propose some methodological considerations for each of the five general research stages (i.e. study design and preparation, sampling and recruitment, informed consent, data collection, analysis and reporting), focusing on what may be different or useful when researching remotely, and using examples from remote qualitative interviewing in Syria.

#### Design and preparation

##### Research consultation group

Remote research presents specific ethical considerations that requires particular consideration of research governance and who should participate in this governance. Ethics review is intended to act as a safeguard against exploitation of research participants and ensure acceptable research methodology and practice [33]. Particularly in the absence of a legitimate in-country review board, conducting research in insecure settings requires explicit interrogation of positionality, power dynamics, and duty of care. We contend that research should be as participatory as possible to help address some of these limitations. To strengthen governance and participation, we established an independent gender-balanced research consultation group of in-country and diaspora Syrian men and women. All members had expertise in health system governance and use of health services in Syria (e.g. patient perspectives). They provided insights throughout the research process (i.e. study co-design, data collection, analysis, dissemination).

##### Diaspora researchers

Our research team was primarily diaspora and in-country researchers. Recruiting and partnering with diaspora researchers was particularly useful. These researchers grew up in Syria and provided health services during the conflict. First, even though diaspora researchers were based in a Western academic institution, their deep connections to the studied country strengthened research access, methods and interpretation, contributing to countering historic exploitation by promoting multidirectional knowledge flows between endogenous and global expertise [34]. Second, diaspora researchers were fluent in English and Arabic. Knowledge of colloquial Syrian Arabic particularly facilitated translation of research tools and conduct of interviews in participants’ dialect. Third, ongoing engagement with the country helped ensure topics were culturally and conflict-sensitive and that questions were relevant and could be rephrased colloquially. Lastly, they were able to access participants through existing personal and professional networks. This was particularly important in establishing trust between participants and the research team. As a participant in research on sexual harassment noted: “*We need someone to deliver our voices and report the situation here. We have faith in someone like you* …” (i.e. a former insider). Researchers with close ties to study settings can use their networks to identify and recruit a range of participants and subsequently increase data richness; (ii) purposefully avoid sampling participants who primarily voice propaganda for particular parties; and (iii) be able to reach ‘ordinary people’ (e.g. service users). However, it should be noted that researcher stance and being labelled an ‘insider’ can also be problematic for researchers during conflicts such as the one in Syria, in which an activist stance can reduce participation or increase risks among those in government-controlled areas, while impartiality – the normative academic socio-political stance – can limit access in other regions and even be interpreted as endorsing a government identified as illegitimate by many in civil society. Relatedly, access through networks must be handled carefully in insecure and conflict-affected settings to avoid endangering participants or leaking sensitive contact information.

##### Psychotherapeutic support

Given the potentially isolating nature of remote research, considerations for remote psychotherapeutic ‘first-aid’ support for participants, transcriptionists, and researchers if topics are potentially difficult or traumatic are important. We recruited a UK-licenced Arabic-speaking psychotherapist to provide on call psychological grounding sessions that we offered free to participants exhibiting distress during interviews. For example, sensitive issues touching upon experiences of personal losses and sexual abuse in the community arose in interviewing a female health-worker, triggering her emotional distress. The researcher had been trained in psychological first aid and drew on active listening skills, paused the interview, and created space for silence. Afterwards, the researcher organised sessions between the psychotherapist and the participant. However, only one session took place before government bombardment of the participant’s city forced her to evacuate and ended her efforts to engage.

Clearly, we need to make this more effective and examine why many participants choose not to engage. For example, ongoing stigma associated with mental health and psychotherapy in Syria, combined with its lower relative priority compared with daily physical insecurities (e.g. food, bombings) likely reduced engagement. Additionally, it is probable that a trained Arabic-speaking Muslim psychotherapist, who is from a different sociocultural context (i.e. Sudanese, living in London), is an important start but not sufficient for participants not to experience this professional as an outsider. Our experiences raise important questions about the limits of endogenous translations of dominant Western psychosocial conceptualisations of trauma and suffering in intercultural contexts, and how these can be made to provide more than linguistic and post-hoc analytical value that often seems overly simplistic [35].

#### Sampling and recruitment

##### Snowballing

Our sampling and recruitment strategies were similar to in-person approaches, with more emphasis on snowballing of researchers’ and participants’ social and professional networks. Snowball sampling is used commonly to research populations labelled ‘hard to reach’ [36]. We contacted professional contacts who fit recruitment criteria and asking each to suggest 1–3 potential participants. We were careful to rely initially on a variety of individuals and social groups to suggest participants to reduce sampling or selection bias. We encouraged ‘cross-category’ suggestions, in which service-users suggested one or more providers for interview, but avoided asking providers to recommend specific service-users to reduce potential selection bias.

In a study of COVID-19 perceptions in displaced communities, we worked with community health-workers to recruit potential participants living in displacement camps in northwest Syria [11]. Researchers followed-up via WhatsApp, introducing themselves, further describing the study, and sharing information sheet and consent forms. This approach achieved very few responses. This was due partially to: (i) the lack of shared acquaintances between participants and researchers, and consequent lack of trust; and (ii) formal and possibly intimidating framing, including mentioning interviews, a distant UK university, and a 3-page information sheet. We changed our approach by dividing messaging into sequential stages and framing it less formally. First, rather than sending all information immediately, we started with a greeting. Second, when participants replied, researchers introduced themselves - avoiding formal titles, e.g. ‘Dr’ - and explained how they obtained potential participants’ WhatsApp number. Third, if participants replied and expressed interest a simple explanation of study objectives and invitation to participate were sent, including that it would be informal, recorded, confidential, and for research purposes (to avoid confusion with media interviews). Researchers immediately followed this by sending the information sheet and consent form. This phased approach yielded much better engagement, with almost all replying to messages and most agreeing to participate. We argue that when prior personal connections with potential participants do not exist, these must be established first with messages kept short, simple, and informal.

##### Diversity

Participant diversity was improved by recruiting for a range of obvious characteristics (e.g. gender, age, place and type of residence - rural/urban/camp/community, occupation, professional seniority). For this to be meaningful, key characteristics were sampled across all settings. For example, since women were more difficult to access, they were sampled across all relevant settings (e.g. geographical, occupation). To address underrepresentation of women in the conflict literature, we ensured a balance by purposefully sampling at least 50% women participants. To help with recruiting and interviewing women, a diaspora Syrian woman researcher organised and conducted the majority of interviews with women. This was very helpful, though male researchers were able to interview women participants successfully when topics were less gender sensitive.

#### Informed consent

##### Written versus verbal

The choice between written and verbal informed consent should be guided by ethics committee requirements and interviewee preferences [33]. Remote written informed consent can be challenging, though not impossible, due to the lack of printers and scanners in many settings. For most of our studies, after an introductory email, message, or voice/video call, study information sheet and consent form were sent via email or internet call application (e.g. WhatsApp) according to the potential participant’s preference.

For a potentially sensitive study of gender-based violence (GBV) during the COVID-19 lockdown in Syria, we used a very different approach to access and consent. First, we contacted an employee at a women’s empowerment centre providing GBV support to affected women. Second, after obtaining permission from centre staff, this field officer acted as a facilitator between researchers and potential participants. She conducted informed consent procedures, scheduled interviews, and provided contact numbers for participants. This helped minimise personal data sharing and maintained the centre’s standards of safety and privacy. As the first contact between researcher and participant was at the interview itself, being introduced through a trusted intermediary was key.

##### Recording consent

We have used several approaches to record consent, depending on recruits’ resources. Those with access to printers and scanners were able to print, sign, scan and send consent forms by email or call app. Those with printer but no scanner access were asked to photograph their signed consent form with a phone camera and return by email or WhatsApp or submit an e-signature. Those without access to either, but with pen/pencil and paper were asked to write the following: “I agree to the conditions in the consent form for study reference XX [“except x” for any conditions they did not agree to]”, then add their name, signature, and date and send a photograph to the researcher. When written consent was not possible, such as for those not comfortable with signing anything or without access to pen and paper (e.g. displacement camp participants) consent was verbally recorded prior to interview mentioning the consent form reference number. Particularly in insecure setting, participants were given the option of signing with a pseudonym to alleviate any data or security concerns. These instructions were clearly written in the consent form and further explained by the researcher to ensure understanding, with conditions for consent numbered to make this process easier.

#### Data collection

##### Topic guides

We developed these in several stages. First, we drafted them in English, the reporting language and spoken by all study team members. Second, the native Arabic speakers translated them into Arabic – participants’ language – after finalisation. Third, Arabic topic guides were back-translated and refined to ensure accuracy and authenticity. Finally, English versions were edited to reflect these refinements as needed. Having researchers with an understanding of social science theory and research methodology, the research topic and country context, and fluency in both English and participants’ language was critical to ensure nuance and meaning were not lost in translation, and guides were both culturally and conflict sensitive.

##### Interpretation

Specific topic guides were developed for different participant categories (e.g. service-users, health-workers) to ensure clarity and relevance of questions. Technical terms for some topics had no broadly-understood equivalents in Arabic, requiring space for testing and errors to adapt the most understandable interpretation. Other terms had different connotations in Arabic, requiring further explanation and vigilance from researchers to avoid misunderstanding. For example, the term legitimacy has religious connotation in Arabic that would not have been appropriate for research on good governance [12]. Arabic topic guides were written in formal Arabic, but questions were asked in colloquial Arabic. This was done for several reasons: First, spoken formal *fus’ha* Arabic is not used in daily life and would feel abnormal and potentially uncomfortable in conversation. Second, colloquial Arabic is not used for writing formal documents and thus not subject to grammar and spelling rules, which differ by person. Therefore, writing in formal Arabic and speaking colloquially provided a reasonable balance of researcher time and is widely practiced. It allowed shared understanding of topic guides among researchers while allowing freedom to phrase questions in different colloquial Arabic dialects. Other languages have similar written/spoken and dialectic differences within countries and regions that must be accommodated in research.

##### Scheduling

Time differences, different weekends/holidays, and punctuality perceptions required consideration when scheduling interviews. Participants sometimes suggested early-morning or late-night times due to conflicting commitments, lack of privacy, security concerns, or internet access. For example, some interviews were scheduled at 06:00 UK time (08:00 Syria time) or 23:00 UK time (01:00 Syria time). This required substantial flexibility from researchers, with flexible and home-working arrangements helping considerably. However, balance between obtaining interviews and maintaining researcher effectiveness must be considered. Time perceptions were different between Syria-based participants and UK-based (Syrian-diaspora) researchers. When contacted, most Syria-based participants were happy to be interviewed the same day or sometimes immediately. Interviews arranged further ahead than the next day were usually missed, with participants not attending and saying they forgot. Some were not able to set a specific time for an interview, instead generalising to “evening” or, when asked to be as specific as possible, saying “after *ishaa* prayer.”

##### Call apps

Interviews were conducted using phone or free internet call applications at times suitable for participants. Where possible, participants chose the call application they preferred, from among sufficiently secure apps, as they had better knowledge of what worked for them given internet constraints in Syria. Choice was affected by factors including familiarity with the technology, security level, voice quality, and internet connection. Table 1 lists the range of possible apps for use in Syria and other countries. Some were perceived as more secure (e.g. Telegram), others work with poor internet (e.g. Messenger), while most people preferred what they used frequently (e.g. WhatsApp). Researchers used encrypted apps where possible and provided guidance on security and any data concerns to participants. Interview length also varied considerably, from 20 min to 2 h with service-users tending to have short interviews and health providers having longer ones, though most lasted approximately 45 min. When the internet connection deteriorated during an interview, researchers consulted participants on the best approach. Sometimes waiting a few minutes was sufficient and sometimes interviews had to be rescheduled for another time or day. On rare occasions, bad internet connections was unavoidable, with longer interviews, repeated questions and answers, skipped questions, and loss of meaning/sentences having to be accepted. Interview recordings were encrypted for security. Recording was usually done using a separate device, e.g. phone or tablet voice recording application, though sometimes built-in recording features were used e.g. Skype.

**Table 1:** Comparison of common internet call applications for remote research

App	Pros	Cons
Facebook Messenger	• Widely available/popular. • Works with poor internet connection. • Allows 50 people in a call (six visible on screen). • End-to-end encryption and secret conversations feature to secure messages, but it must be activated before use. • Messages can be set to self-destruction after a certain period of time (between five seconds and 24 h).	• Linked to personal Facebook profile, so not anonymous. • The new desktop version only allows eight participants in a video call. • A sizable security downside is that Messenger calls are not encrypted by default, unless you turn on the “secret conversation” feature. This means a copy of the message remains on Facebook’s servers if the feature is not activated. • Facebook’ practices around privacy has been a concern.
Google Hangouts	• Simple to use on both mobile and desktop. • Requires a Google account to set up a meeting. • Available free on both iOS and Android. • It does encrypt hangouts conversations, but does not use end-to-end encryption — instead, messages are encrypted “in transit. (This means that they are only encrypted between the device and Google’s servers. Once they are on a server, Google has complete access to them). • Allows for group up to 25 people. • It does not need to be installed on devices, as sending over an invitation or link is enough.	• Google Hangouts is riddled with privacy and security concerns. Though the calls are encrypted, Google makes use of various user metadata whenever it can. • Google Hangout is not as popular as other apps. • Images sent via Hangouts are shared through public URLs, meaning that virtually anyone (who knows a thing or two about URLs) can view them.
Signal	• The gold standard of messaging security app. • One of the most secure messaging apps on the market, the company does not collect customer data. • Messages and voice and video calls are end-to-end encrypted by default. Not even the owner company can decrypt the messages. • Signal is available for Android and iOS mobile devices. • It allows to make both sent and received messages “disappear” after a certain amount of time has elapsed. • The app also allows a password to lock it.	• Not widely used • Currently, video calls are only one-on-one. • Only available on mobiles.
Skype	• Skype is widely compatible and pre-installed on some computers. • You get maximum 4 h/ session for free and maximum video meeting size of 50 people. • Encryption is automatically activated when calling. • There is a version for business called *Skype Business* which is pretty cheap, and you get 250 video meeting slots and extra security.	• Not widely used in Syria. • Works poorly with poor internet. • Works poorly on mobile phones. • Skype is owned by Microsoft, which is rumoured to have collaborated with intelligence agencies to circumvent user privacy. • When using regular telephone calls with Skype (where you do have to pay normal calling fees to call actual mobile or landline phones), the encryption doesn’t apply.
Telegram	• End-to-end encryption with a feature called “Secret Chat” to protect messages. • Passcode Lock, a four-digit code to prevent intruders from accessing the messages. • Self-destructing messages, (for Secret Chats only) that will delete private text messages and media within a pre-set time limit. • Remote logout, because it offers log into from numerous devices at the same time (web, PC, tablet, smartphone, etc.), so the app offers the ability to log out of other sessions from the current device is used through the Settings menu. • Account self-destruct, an inactive account for a certain amount of time (six months being the default) with completely wiping clean all of the messages and media.	• Not widely used. • The encryption feature is not default; it must be turned on manually before using.
WhatsApp	• Most used app in Syria/popular. • Allows for groups of up to eight people. • WhatsApp Voice and video calls and messages are end-to-end encrypted by default, which increased security. • WhatsApp also has a “*Verify Security Code”* screen in the contact info screen that allows the user to confirm that the calls and messages are end-to-end encrypted. • It is available free on both iPhone and Android. • The only time of which the message is kept on a WhatsApp server is the period after sending it and before it is delivered to the receiver. If it cannot be delivered for some reason, then the message will be deleted from the server after 30 days.	• Linked to mobile number, potentially hindering security. • Used for personal communication so potentially less anonymous (e.g. if profile picture used). • Only allows up to four people in a group video chat. • Though content is encrypted, WhatsApp is owned by Facebook, a company which makes excessive use of user data.
Zoom	• Widely used globally in the current COVID19 epidemic. • Allows for groups of up to 50 people with screen sharing and breakout rooms. • It has a built-in recording feature. • Zoom allows the most participants of any; on a free and basic paid plan, up to 1000 people can join a single call. • Allows scheduling meetings ahead of time. • There is a “Waiting Room” feature, so the meeting’s host can see potential participants before they join the meeting. • It does not need to be installed, sharing the 10-digit personal meeting ID or send over a Zoom link is enough.	• Still relatively new and unfamiliar. • Not the simplest service to use; free version is limited to 40 min. • More computer friendly than phone friendly. • Potential security issues. Zoom’s encryption was discovered to not be as strong as expected. • Interlopers were also found disturbing – or “*Zoom-bombing*” – private meetings. However, this has been quickly tackled by requiring passwords for every meeting and turning on the Waiting Room feature by default.

Adapted from: https://www.wired.co.uk/article/best-video-conference-apps; https://www.avg.com/en/signal/pros-and-cons-of-video-chat-apps; https://www.avg.com/en/signal/secure-message-apps (Accessed 20 October 2020)

##### Group interviews

Remote group interview facilitation is challenging in low-income and insecure settings, particularly if participants are located separately, as internet is rarely strong enough to allow for video calls. Although we have not yet been able to conduct methodologically rigorous focus-group discussions, we found that facilitated group discussions provided rich data. Early learning from this approach suggests that in insecure settings discussion groups work best if participants already know each other and are comfortable sharing experiences and information. While this may reduce differences in viewpoints, it allows more open and frank responses to topics. Timing and participants’ locations are crucial, requiring considerable researcher flexibility. For example, if health-workers participating in a group discussion come from the same health facility, the internet connection works better if they sit in a room together and share a device. However, when participants must remain separated – such as for the COVID-19 pandemic or security restrictions, more focused sessions with simpler questions are required. Some challenges were exacerbated, such as issues of sound lag, noise interference, and difficulties distinguishing what people are saying, meaning that more time and patience was required than for in-person group discussions. Data saturation was determined in the same way as for in-person data collection, such as by using the saturation grid described by Fusch and Ness [37].

#### Analysis and reporting

While analysis and reporting do not differ significantly from that for in-person research, two related issues that may require consideration are transcription and translation.

##### Transcription

Many colloquial dialects, such as spoken Syrian Arabic, are considerably different from formal written forms. It was thus particularly important that audio data were transcribed by transcriptionists fluent in the dialect spoken by participants. Similarly, transcripts were written in spoken dialect to ensure meaning and nuance were not lost. Therefore, we hired two Syrian transcriptionists to transcribe anonymised encrypted audio files (e.g. with any identifying information removed) and then delete their copies of audio and transcript files once shared with the research team.

##### Translation

Whether or not to translate data prior to analysis is a key consideration. There are benefits and challenges to either approach, which we considered carefully before choosing to analyse transcript data in colloquial Syrian Arabic to maintain authenticity and nuance. Another benefit was that this saved considerable time and money. A significant challenge in our multi-language team was that not all team members were sufficiently fluent in Arabic, which potentially weakened cross-checking, supervision, and capacity-building. A second challenge was that common data management software does not function well with Arabic data. For example, we found that while NVivo could be used for Arabic transcripts with some adaptations (e.g. manipulating document formatting before uploading, coding whole lines rather than one word), advanced features were limited or impossible (e.g. word searches or word ‘clouds’ due both to lack of recognition of Arabic script and the need to use colloquial language).

##### Dissemination

While remote research methods do not limit or prevent traditional dissemination methods, we found several advantages of remote methods in disseminating research findings, particularly for participant follow-up and reporting back to communities. Having online connections with participants allows for easy and direct dissemination of research findings to them. However, the difference in research language (e.g. English) and participant language (e.g. Arabic) can be an important obstacle. Translation of key research outputs, such as publications, back into participants’ language should be considered as additional research outputs and budgeted for in research proposals. Similarly, any dissemination events should consider both languages. Remote approaches are an obvious asset in sharing findings with relevant stakeholders across the world. Familiarity with online platforms for research makes them easier to use for dissemination (e.g. conducting multilingual webinars via zoom, publishing in open access journals, and promoting research findings through the social media providers most widely used in the settings in which the research was conducted) [38].

## Conclusion

Our experiences conducting remote qualitative research in Syria highlight specific ethical, methodological, and logistical issues that require consideration before conducting remote research. We acknowledge that remote research is not always the answer to researchers’ access constraints. For example, accessing communities remotely could further marginalise individuals with poor or no internet access. However, when conducting research remotely is an appropriate approach to answer research questions and is appropriate for targeted participants, it can be a rewarding way to explore new areas of research and gain insights from communities who might otherwise be rendered invisible by more mainstream face-to-face data collection methods. In particular, our experience shows that an approach that combines remote ways of interacting with conflict-affected communities, adherence to academic rigour, and efforts to increase participation and engagement, can lead to more locally-appropriate and conflict-sensitive research encounters. Key to the success of this approach is the quality of the virtual relationships researchers can develop and maintain without face-to-face interactions and with limited physical time in the country. For this type of engaged remote research to be effective, lead researchers must have a meaningful connection with the area in which they are conducting research, even if this might not be possible for the whole team. Importantly, connections between researchers and participants will be (re) negotiated throughout the research process. Connections to the context studied need to be foregrounded in analysis and dissemination processes, as illustrated by the importance of fluency in colloquial Arabic to understand nuances in meanings in Syria, and of personal experiences in-country to understand what can or cannot be said publicly in different circumstances. This approach, when added to the ongoing dialogue around overdue efforts to decolonise global health research, can help both to reduce the chance that remote research will be extractive and exploitative and to strengthen the quality of interpretation and value of outputs.

## Data Availability

The data generated and analysed during this study is not publicly available due to participant confidentiality.
